# Supplementary material for: Generation of Hydrogen Peroxide and Downstream Protein Kinase D1 Signaling Is a Common Feature of Inducers of Pancreatic Acinar-to-Ductal Metaplasia
Source: Antioxidants (Basel). 2022 Jan 8;11(1):137. doi: 10.3390/antiox11010137 (PMC8772746; doi:10.3390/antiox11010137)
Supplement: Supplementary file 1 [file antioxidants-11-00137-s001.zip › antioxidants-1515568-supplementary.pdf]

Supplemental Figure S1

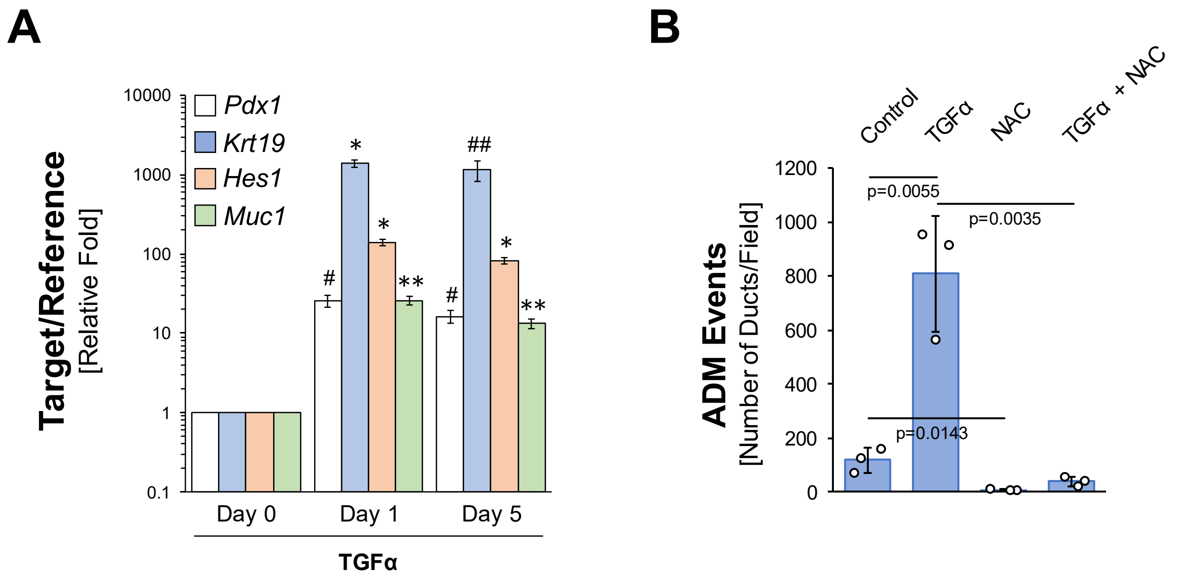

Supplemental Figure S1, Supplement to Figure 1. **A: TGF*α* induces RNA expression related to ADM.** Mouse pancreatic primary acinar cells were isolated and seeded in 3D collagen culture. At indicated days, cells were isolated from the collagen and analyzed by quantitative PCR for expression of ADM related genes. **B: TGF*α*-induced ADM is blocked by NAC.** Mouse pancreatic primary acinar cells were isolated and seeded in 3D collagen culture. As indicated, cells were treated with NAC (5 mM) and ADM was induced with TGF*α* (50 ng/ml). Ducts formed were quantified as indicated in the Methods section. **A, B:** All experiments shown were performed in triplicates for at least 3 times and obtained similar results each repeat. Statistical analysis between two groups was performed using t-test. A p value of 0.05 was considered statistically significant and values are included in the graphs.

Supplemental Figure S2

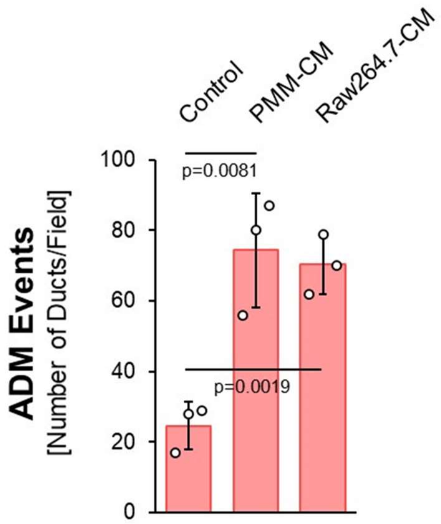

Supplemental Figure S2, Supplement to Figure 2. **Macrophage-conditioned media induces ADM.** Mouse pancreatic acinar cells were isolated, seeded in 3D collagen culture and then stimulated with conditioned media from primary peritoneal mouse macrophages (PPM) or Raw264.7 macrophages. Ducts formed were quantified as indicated in the Methods section. Statistical analysis between two groups was performed using t-test. A p value of 0.05 was considered statistically significant and values are included in the graph.

Supplemental Figure S3

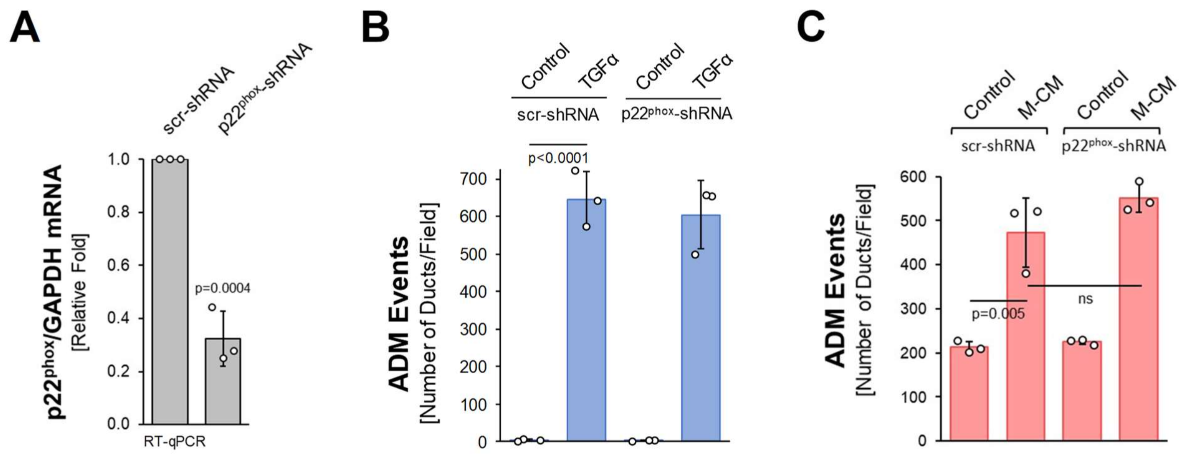

Supplemental Figure S3, Supplement to Figure 3. **The knockdown of p22phox does not affect ADM. A:** Expression of p22phox was determined by RT-qPCR as described in the Methods section 48 hours after infection. The experiment was performed in triplicates for 3 times and obtained similar results each repeat. Statistical analysis between the two groups was performed using t-test. A p value of 0.05 was considered statistically significant and the value is included in the graph. **B, C:** Mouse pancreatic acinar cells were isolated, lentivirally-infected with control or p22phox-targeting shRNA and seeded in 3D collagen culture. Cells then were treated with 50 ng/ml TGFα (B), M-CM (C) and respective controls. Ducts formed were quantified as indicated in the Methods section. All experiments shown were performed in triplicates for at least 3 times and obtained similar results each repeat. Statistical analysis between two groups was performed using t-test. A p value of 0.05 was considered statistically significant and values are included in the graphs.

Supplemental Figure S4

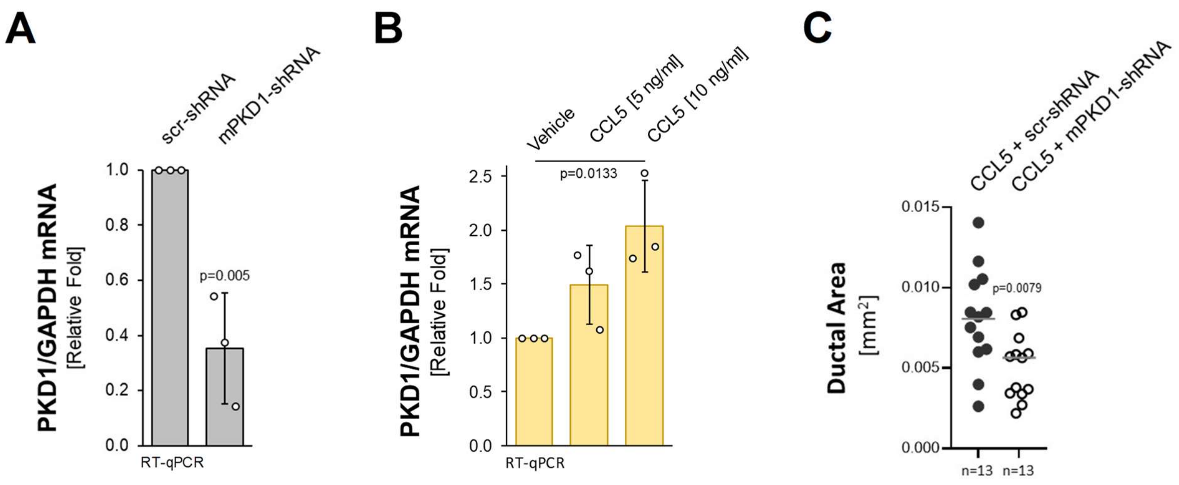

Supplemental Figure S4, Supplement to Figure 4. **Role of PKD1 in ADM. A:** Verification of mPKD1 knockdown. Expression of mouse PKD1 was determined by RT-qPCR as described in the Methods section 48 hours after infection. The experiment was performed in triplicates for 2 times and obtained similar results each repeat. Statistical analysis between the two groups was performed using t-test. A p value of 0.05 was considered statistically significant and the value is included in the graph. **B:** PKD1 upregulates expression of CCL5. Mouse pancreatic acinar cells were isolated and stimulated with indicated dose of CCL5 for 48 hours. Cells were collected and analyzed by quantitative PCR for expression of PKD1. The experiment was performed in triplicates for 3 times and obtained similar results each repeat. Statistical analysis between two groups was performed using t-test. A p value of 0.05 was considered statistically significant and the value is included in the graph. **C:** PKD1 knockdown affects duct size of ADM. Samples from Fig. 4D. Ducts formed after induction of ADM were analyzed for their ductal area using ImageJ.

Supplemental Figure S5

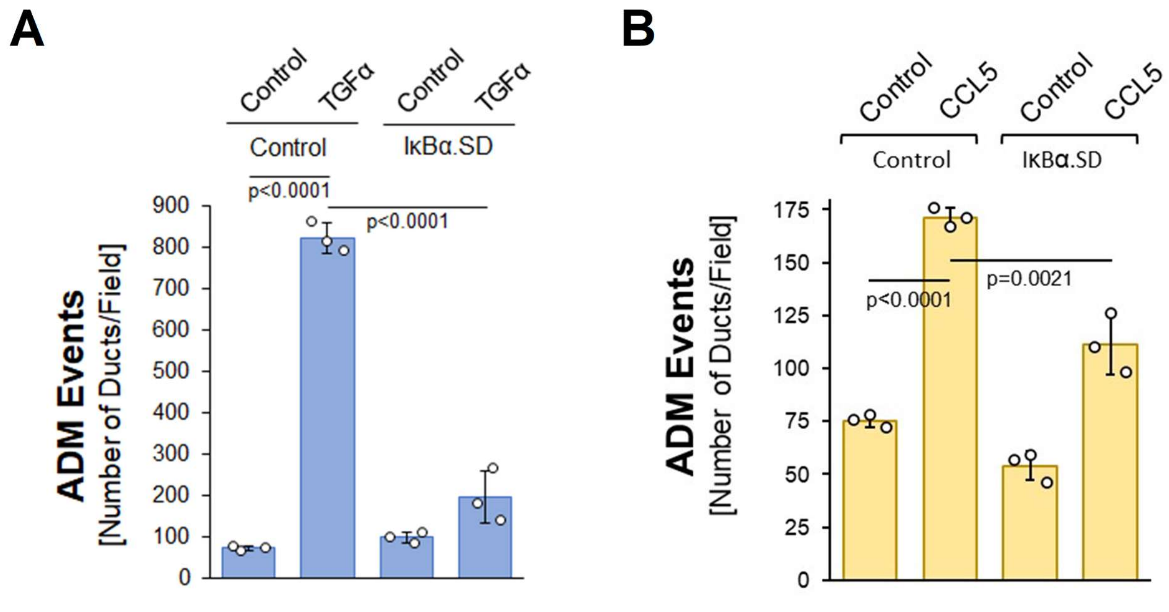

Supplemental Figure S5, Supplement to Figure 5. **TGFα- and CCL5-induced ADM is inhibited by super-dominant IκBα. A:** Mouse pancreatic acinar cells were isolated, adenovirally-infected with control virus or superdominant IκB*α* (IκB*α*.SD) and seeded in 3D collagen culture. ADM was induced with TGF*α* (50 ng/ml) and ducts formed were quantified as indicated in the Methods section. **B:** Mouse pancreatic acinar cells were isolated, adenovirally-infected with control virus or superdominant IκB*α* (IκB*α*.SD) and seeded in 3D collagen culture. ADM was induced with CCL5 (50 ng/ml) and ducts formed were quantified as indicated in the Methods section. **A, B:** All experiments shown were performed in triplicates for at least 3 times and obtained similar results each repeat. Statistical analysis between two groups was performed using t-test. A p value of 0.05 was considered statistically significant and values are included in the graphs.
